# APPTEST is a novel protocol for the automatic prediction of peptide tertiary structures

**DOI:** 10.1093/bib/bbab308

**Published:** 2021-08-14

**Authors:** Patrick Brendan Timmons, Chandralal M Hewage

**Affiliations:** UCD School of Biomolecular and Biomedical Science, UCD Centre for Synthesis and Chemical Biology, UCD Conway Institute, University College Dublin, Dublin 4, Ireland; UCD School of Biomolecular and Biomedical Science, UCD Centre for Synthesis and Chemical Biology, UCD Conway Institute, University College Dublin, Dublin 4, Ireland

**Keywords:** neural network, machine learning, peptide, structure prediction, NMR, crystallography

## Abstract

Good knowledge of a peptide’s tertiary structure is important for understanding its function and its interactions with its biological targets. APPTEST is a novel computational protocol that employs a neural network architecture and simulated annealing methods for the prediction of peptide tertiary structure from the primary sequence. APPTEST works for both linear and cyclic peptides of 5–40 natural amino acids. APPTEST is computationally efficient, returning predicted structures within a number of minutes. APPTEST performance was evaluated on a set of 356 test peptides; the best structure predicted for each peptide deviated by an average of 1.9Å from its experimentally determined backbone conformation, and a native or near-native structure was predicted for 97% of the target sequences. A comparison of APPTEST performance with PEP-FOLD, PEPstrMOD and PepLook across benchmark datasets of short, long and cyclic peptides shows that on average APPTEST produces structures more native than the existing methods in all three categories. This innovative, cutting-edge peptide structure prediction method is available as an online web server at https://research.timmons.eu/apptest, facilitating *in silico* study and design of peptides by the wider research community.

## Introduction

Interest in peptide therapeutics has grown significantly in recent years, motivated in part by the many advantages peptides possess compared to traditional small molecule chemical drugs [1, 2]. Peptide therapeutics are more selective, specific and efficacious than small molecule drugs and are degraded to amino acids, which are less likely to exhibit undesirable drug–drug interactions. Furthermore, peptides are less likely to accumulate in tissues due to their short half-life, are less susceptible to the development of drug resistance and are cost effective to produce [3–6].

Peptides are an important and ancient element of the immune response of all life forms, with host defense peptides having been identified in all species, and found to be highly conserved in vertebrate, insect and plant genomes [7, 8]. Peptide therapeutics are typically short, with a sequence length of 5–30 amino acids, and were initially isolated from plant or animal secretions but are now also obtained from chemical [9], genetic [10] and recombinant [11] libraries. Combined, these libraries present a largely unexplored chemical space. Peptides have been identified with antibacterial, antifungal, antiparasitic, antiviral and anticancer properties. A limited number of peptide drugs are currently available for treatment, including bacitracin, boceprevir, enfuvirtide and leuprolide, for treatment of pneumonia, hepatitis-C, HIV and prostate cancer, respectively. Therapeutic peptides have been found to possess $\alpha $-helical, $\beta $-sheet and extended conformations in the presence of membrane or membranomimetic environments [12–17].

The quantity of sequence data available from sequencing experiments has grown rapidly in recent years. The quantity of sequences with experimentally determined tertiary structures, however, is lagging behind, as determining structures experimentally is a cost and time-intensive task. Given that peptides’ bioactivities are dependent on their structure, being able to easily obtain the peptides’ tertiary structure from their primary sequence would facilitate an acceleration of the peptide drug design pipeline.

The prediction of protein structures from their primary sequences represents one of the most challenging problems in bioinformatics today. Many attempts have been made at solving the protein structure prediction problem, with many software applications having been developed for this purpose, including I-TASSER [18], Rosetta [19], HHpred [20], NovaFold and most notably AlphaFold 2 [21] which recently performed excellently in the CASP 14 experiment. The prediction of peptide structures, which are distinguished from proteins by their short sequence length, presents a similar challenge, which has not received the same attention as the former, with only a limited number of programs developed for the purpose of predicting peptide tertiary structure. While programs like AlphaFold 2 can utilize co-evolutionary information to predict inter-residue contacts, the same is not always possible for peptides. A number of attempts have been made in the past at accurate prediction of peptide tertiary structure. Geocore, an *ab initio* filtering algorithm, was developed for finding native-like structures in small ensembles of conformations [22]. Later, PEPstr was developed for the prediction of peptide tertiary structures from predicted beta-turn and secondary structure information [23]. PEPstr has since been superseded by PEPstrMOD, which expands the scope to include cyclic peptides and peptides with non-natural residues [24]. Nicosia and Stracquadanio [25] employed a generalized pattern search (Gps) algorithm, which uses search and poll to find peptide conformation global energy minima. PepLook explores the peptide conformational space using a Boltzmann stochastic algorithm [26–28]. At a similar time, Maupetit *et al.* developed PEP-FOLD, which has been updated multiple times [29–33]. The most recent version combines a structural alphabet with a hidden Markov model. Finally, Narzisi *et al.* [34] employed a multi-objective evolutionary algorithm for the exploration of the peptide conformational space.

Machine learning techniques, including deep learning, have previously been applied to other bioinformatic problems: DeepPPISP for the prediction of protein–protein interaction sites [35], SCLpred and SCLpred-EMS for protein subcellular localization prediction [36, 37], CPPpred for the prediction of cell-penetrating peptides [38], HAPPENN for the prediction of peptide hemolytic activity [39], ENNAACT for the prediction of peptide anticancer activity, [40] and ENNAVIA for the prediction of peptide antiviral activity [41]. Indeed, deep learning has been applied to the prediction of protein secondary structures [42–44]. Herein, we describe APPTEST, a novel protocol for the automatic prediction of peptide tertiary structures. APPTEST utilizes one-dimensional gated residual convolutional neural networks for the prediction of distance and dihedral angle restraints, which are then input to traditional NMR structure determination methods to obtain a final ensemble of model structures.

## Methods

**Figure 1:**
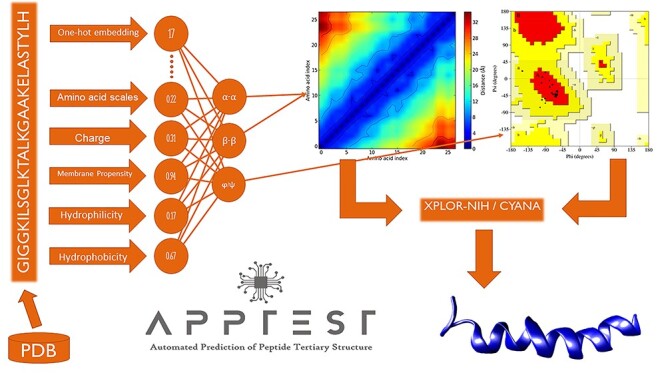
Graphical summary of the APPTEST protocol. Structures and corresponding peptide sequences are extracted from the Protein Data Bank; eligible sequences are retained to form the APPTEST dataset. Peptide sequences are described on a per-amino acid basis, using one-hot embedding and amino acid scales. Neural networks are trained using the described sequences as inputs and inter-residue distance and torsion angles as the prediction targets. Predicted structural restraints are used as inputs to modeling programs XPLOR-NIH or CYANA, which use molecular dynamics simulations to yield an ensemble of predicted structures.

The APPTEST protocol combines deep learning methodology with traditional NMR structure determination methods to predict peptide tertiary structures. Figure 1 provides a graphical summary of the APPTEST protocol, which is further detailed henceforth.

### Dataset

The proper construction of a reliable dataset is an important step in a machine learning endeavor. Neural networks’ performance scales with the size of the dataset available for training; it is important, therefore, to construct a dataset encompassing as many peptide structures as possible from multiple sources.

PDB structure codes were sourced from multiple peptide databases: DBAASP [45, 46], APD3 [47], ADAM [48] and DRAMP [49]. PDB structure codes were also extracted from the datasets of PEPstrMOD [23] and PEP-2D [50]. Finally, the RCSB PDB [51] was also searched for structures with a chain length between 5 and 40 amino acids.

Only structures with a sequence length between 5 and 40 were considered. To prevent the classifier from overfitting to the training data, the dataset’s sequences were internally redundancy reduced using CD-HIT [52–54], with a sequence identity cut-off of 0.9. NMR structures with an internal backbone RMSD greater than 2.50 Å were excluded.

### Model validation

It is critically important to thoroughly validate machine learning models. Ten-fold cross-validation and validation by an external test set were employed to evaluate the performance of APPTEST. A total of 2265 experimentally obtained peptide structures were used for model training and internal 10-fold cross-validation. The models trained under cross-validation were ensembled and evaluated with the external test set, which consists of 356 previously unseen, redundancy reduced peptide sequences and their corresponding experimentally obtained peptide structures.

### Structure analysis

The Biopython [55] module Bio.PDB [56] was used to retrieve peptide structures from the PDB and to calculate inter-residue distances, dihedral angles and root mean square deviation (RMSD) values.

### Peptide representation

A total of 186 amino acid scales were extracted from the AAindex [57] and used to construct the matrix $A$, of shape (21, 186), where the 1st 20 rows correspond to the twenty proteinogenic amino acids, the last row corresponds to the non-natural amino acids without known AAindex-scaled properties and the 186 columns correspond to the 186 amino acid indices. $A^{T}$ is scaled using the standard scaler, is dimensionality reduced with principal component analysis [58] and scaled so its values have minimum and maximum values of zero and one, respectively. The final matrix $A$ has shape (21, 15).

Each peptide can be represented by one-hot encoding its primary sequence to give a vector $h$, where 0 represents an empty position, 1–20 encode the natural, proteinogenic amino acids and 22 encodes a non-natural amino acid residue. Similarly, the one-hot encoding can be represented by a sparse matrix $P$ of shape (50,21) exists, where $P_{ij} = 1$ if the amino acid at sequence position $i$ is the $j$th of the 21 types of amino acid. Information about cyclic restraints can be encoded in a sparse matrix $C$ of shape (50,50), where $C_{i,j}=1$ if a cyclization exists between the $i$th and $j$th residues. Finally, a matrix $S$ of shape (50,15) which describes the peptide’s amino acids’ physicochemical properties can be defined as follows:

(1) \begin{align*}& S = P \cdotp A. \end{align*}

Additionally, two masks can be defined. A vector *m*, of length 50, and a matrix *M*, of shape (50,50). $m_{i} = 1$ if the $i$th position of the peptide sequence space is occupied. Similarly, $M_{i,j} = 1$ if the $i$th and $j$th positions of the peptide sequence space are occupied.

### Data augmentation

In order to artificially increase the quantity of data available, data augmentation is employed. Specifically, the peptides’ inputs to the neural networks are shifted along the input frame of length 50. During training, each peptide is represented as 20 samples, each randomly shifted along the input frame. During blind prediction, each peptide is represented as *n* samples, where *n* is 50-(peptide length)+1. The neural networks’ outputs are shifted back to the original frame and averaged.

### Neural network architecture and implementation

Keras with a Tensorflow [59] backend was used for the construction and training of the neural network. A randomized grid search strategy was employed for the identification of the optimal neural network architecture and hyperparameters.

First, $h$ is input to an embedding layer with a dense embedding dimension of 12. Each row of the dense embedding is then multiplied by the mask vector $m$, resulting in a final tensor of shape (50,12). This tensor is concatenated with $A$ and $C$ to yield a final tensor of shape (50, 77), which is input to a one-dimensional convolutional layer, with 128 filters and a window width of 7. This is followed by batch normalization layer, the rectified linear unit activation function, a one-dimensional spatial dropout layer and two residual gated convolutional blocks.

Each residual gated convolutional block consists of three one-dimensional gated convolutional layers [60], which also have 128 filters and a window width of 7. The 1st two are followed by a batch normalization layer and a rectified linear unit activation function, and the final has a spatial dropout applied to it. The output of the spatial dropout layer is added to the block’s original input, batch normalized, activated with the rectified linear unit and has another spatial dropout layer applied. The output of the 2nd residual gated convolutional block is connected to a fully connected layer with 1024 nodes, which is followed by a batch normalization, a rectified linear unit activation and a dropout layer. This layer is connected to three output layers, which have 2500, 2500 and 200 nodes, respectively. The 1st two are activated with the rectified linear unit, and the third is activated with the hyperbolic tangent function. When reshaped, and multiplied with their respective masks, these output layers correspond to the C_$\alpha $_-C_$\alpha $_ and C_$\beta $_-C_$\beta $_ distances and the $\cos $ and $\sin $ of the peptide’s $\phi $ and $\psi $ dihedral angles.

The mean squared error (MSE) function, commonly used for regression tasks, is employed as the loss function in training the neural network. It is defined as follows:

(2) \begin{align*}& MSE = \frac{1}{N}\sum_{i=1}^{N}[(y_{i} - \hat{y_{i}})^2] \end{align*}

where *y_i_* is the true value of the *i*th sample and $\hat{y_{i}}$ is the predicted value of the *i*th sample.

Adaptive momentum with Nesterov momentum (Nadam) was identified as the optimal optimizer [61].

The neural network was trained for 400 epochs, without stopping criteria. The model with the lowest validation MSE encountered during training was retained for each of the cross-validation splits. The optimal learning rate parameter was found to be 0.001.

### Simulated annealing protocols

#### Distance restraints

$C_{\alpha }-C_{\alpha }$ and $C_{\beta }-C_{\beta }$ distance restraints are derived from the neural networks’ predictions, with the lower distance restraints being calculated as $mean-sd$, and the upper distance restraints being calculated as $mean+sd$. For a peptide with a sequence of length $n$, a total of $2n(n-1)$ distance restraints are generated.

#### Dihedral restraints

The predicted values for each dihedral angle’s $\cos $ and $\sin $ values are averaged, and those average values are used to recover a predicted dihedral angle value. The upper and lower dihedral angle restraints are given as $mean+15^{\circ }$ and $mean-15^{\circ }$, respectively. For a peptide with a sequence of length $n$, a total of $2(n-1)$ dihedral restraints are generated, of which only predictions where both the $\cos $ and $\sin $ standard deviations are below 0.10 are included as dihedral restraints. Consequently, the majority of structural restraints used in the structure determination are distance restraints.

#### XPLOR-NIH protocol

XPLOR-NIH 3.1 is used for simulated annealing and energy minimization, yielding a default of 100 structures [62, 63]. Structure coordinates are initiated from a preliminary structure constructed using PeptideBuilder [64], based on neural networks’ predicted dihedral angles. Upper and lower distance and torsion angle restraints are loaded, and torsion angle dynamics are performed, with an initial temperature of 2025 K and a final temperature of 25 K. A temperature step of 25 K is employed, with a tolerance factor of 100 for the annealing stage. Finally, Cartesian angle minimization is performed.

#### CYANA protocol

Alternatively, CYANA 3.0 is also used for simulated annealing [65]. The upper and lower distance and torsion angle restraints are loaded, and by default, 100 random structures are created, annealed for 20 000 steps and energy minimized for 20 000 steps.

### Performance evaluation

The robustness of the predictions is evaluated by measurement of the backbone RMSD (B-RMSD) between the predicted and experimental structures. Results are reported for both the best-predicted model and the ensemble’s prime model. The choice of the ensemble’s prime model is dependent on the modeling software employed: CYANA models are ordered by target function value, while XPLOR-NIH models are by default ordered by total energy. B-RMSD values are calculated for both the full structure and the rigid core (RC). A structure is considered near native, if the RC B-RMSD to the experimental conformation is <4Å, as previously defined by Thévenet *et al.* [31].

### Rigid core

As NMR model structures can exhibit significant structural diversity, ensemble-level comparisons between the predicted and experimental structures are performed for the peptides’ RCs as well as the full structures. RC regions were calculated using the method described by Maupetit *et al.* [30] A peptide’s RC is defined from the experimentally obtained structure as the set of residues that exhibit a C$\alpha $-RMSD of <1.5 Å.

### Comparison with existing methods

The predictive performance of APPTEST is compared with the existing peptide tertiary structure prediction methods. The structures selected for the comparison are those used for similar comparison tables in the articles describing PEPstr [23], PEPstrMOD [24], PEP-FOLD [29–32] and PepLook [28].

APPTEST is used with XPLOR-NIH to predict 100 structures. The structures are sorted by their total energies. PEP-FOLD 3.5 is used to predict 100 structures. The PEP-FOLD structures are sorted by the sOPEP energy, and all 100 structures are retained. For cyclic peptides, PEP-FOLD 2.0 is used, as recommended by the authors, with the recommended short simulation time employed. PEPstrMOD only predicts a single structure for each query. The simulation time used is 100 ps in a vacuum environment. PepLook does not have an online interface that can be used for structure prediction; consequently, it was not possible to independently evaluate the PepLook method. The results presented are reproduced directly from Beaufays *et al.* [28].

## Results and discussion

### Model validation

In order to comprehensively evaluate the predictive ability of APPTEST, predicted structures were calculated for the 356 peptide sequences of the independent test set. The full set of results is detailed in Supplementary Table 1. A summary of the structural statistics is given in Table 1.

**Table 1:** APPTEST performances on the APPTEST independent test set, using CYANA and XPLOR-NIH for torsion angle dynamics and simulated annealing. B-RMSD values are given for the best-predicted model, the model with the least distance restraint violations and the model with the lowest energy (XPLOR-NIH only). Numbers in brackets are B-RMSD values for the peptides’ RCs only

Program		CYANA		XPLOR-NIH		
LENGTH	N	BEST	RESTR.	BEST	ENERGY	RESTR.
6–12	123	1.85 (1.83)	2.43 (2.41)	1.57 (1.56)	2.30 (2.28)	2.45 (2.44)
13–19	83	2.07 (1.95)	2.63 (2.47)	1.93 (1.80)	2.70 (2.52)	2.71 (2.56)
20–26	44	2.57 (2.33)	3.25 (2.96)	2.39 (2.18)	3.05 (2.74)	3.12 (2.83)
27–33	52	2.21 (2.01)	2.71 (2.48)	2.07 (1.88)	2.69 (2.42)	2.65 (2.41)
34–40	41	2.59 (2.30)	3.00 (2.69)	2.50 (2.20)	3.07 (2.69)	3.36 (3.00)
All	356	2.10 (1.98)	2.65 (2.51)	1.91 (1.79)	2.61 (2.44)	2.70 (2.54)

While APPTEST itself generates distance and dihedral restraints, a molecular modeling program is required for the creation of model structures based on these restraints. Two molecular modeling packages are employed and compared in this work: CYANA and XPLOR-NIH. The former benefits from faster computation times, but requires a license for use, which precludes us from integrating it with our web server. The web server can create the required CYANA-format restraints which the user can use for the creation of structures with their local copy of CYANA or any server that supports CYANA. The latter, while computationally slower, can be downloaded with a license for free for academic use from the authors’ website.

**Figure 2:**
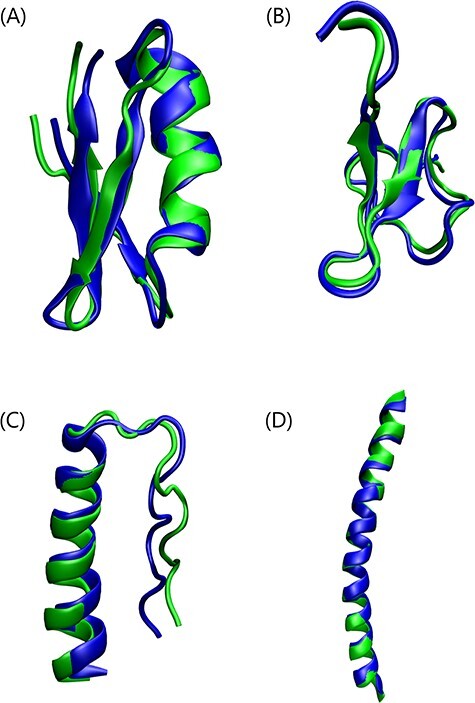
Selection of some of the best APPTEST predicted structures (green) aligned with the peptides’ corresponding experimental conformations (blue). (**A**) Hongotoxin 1 (1hly) is a 39 amino acid mixed-structure peptide, which has both $\alpha $-helical and $\beta $-sheet structures [66]. The best APPTEST structure has a B-RMSD of 1.44 (1.38) Å to the experimental conformation. (**B**) Jingzhaotoxin-XI (2a2v) is a 34 residue peptide with two $\beta $-sheets [67]. The best APPTEST structure has a B-RMSD of 1.46 (1.31) Å to the solution NMR structure. (**C**) Optimized PPa-TYR (6gwx) is a 36 residue rationally designed miniprotein with a polyproline-II helix and an $\alpha $-helix motif [66]. The best APPTEST structure has a B-RMSD of 1.75 (1.74) Å to the experimentally determined structure. (**D**) The hydrophobic analogue of winter flounder antifreeze protein (1j5b) is a 37 amino acid $\alpha $-helical structure. The APPTEST predicted structure with the lowest energy is the best-predicted structure, with a B-RMSD of just 0.86 Å to the experimental conformation [68].

The structure prediction results tabulated for the 356 peptides in Table 1 show that APPTEST is a reliable method for the prediction of structures of peptides of 5-40 amino acids. The performance with both packages is comparable, although the better performance is achieved with the XPLOR-NIH package, with a mean best B-RMSD of 1.91 Å, compared to a B-RMSD of 2.10 Å when using CYANA for the 356 peptides. A mean B-RMSD of just 1.57 Å is achieved for short peptides between 6-12 amino acids in length, rising to just 2.50 Å for longer peptides with between 34 and 40 amino acids. The best RMSD achieved was 0.23 Å for the structure 2mjr, which is 10 amino acids long, while the worst RMSD was 8.38 Å for the structure 2ki0. An RMSD below 3.00 Å is achieved for 84% of the 356 structures tested, and an RMSD below 2.00 Å is achieved for 60% of the structures tested. Furthermore, only 3% of peptides have a best B-RMSD greater than 4.00 Å. A selection of some of the best APPTEST predicted structures is illustrated in Figure 2.

### Comparison with existing methods

To effectively benchmark the predictive performance of APPTEST against PEP-FOLD, PEPstrMOD and PepLook, the structures predicted by each method were compared to the experimental structures and the B-RMSD values were measured. Three benchmark sets of structures are used: short peptides (9-25 aa), long peptides (26-40 aa) and cyclic peptides (10-30 aa). The results are detailed in Supplementary Tables 2–4 and summarized in Table 2.

**Table 2:** Performance comparison summary of APPTEST, PEP-FOLD, PepLook and PEPstrMOD B-RMSD values on short, long and cyclic peptides. Results are reported for the best model and the prime (lowest energy) model, for both the full structure and the RC (given in brackets, where applicable). APPTEST achieves the best performance on more peptides in each class than existing approaches and achieves the lowest mean B-RMSD values. Most notably, considering cyclic peptides of 10-40 aa, APPTEST’s mean lowest energy structure B-RMSD is just 64% that of the next best, PEP-FOLD

	N	L	APPTEST	PEP-FOLD	PepLook	PEPstrMOD	APPTEST	PEP-FOLD	PepLook
			PRIME	PRIME	PRIME	ONLY	BEST	BEST	BEST
SHORT	42	9–25	2.60 (2.24)	2.81 (2.38)	–	4.66 (4.33)	1.96 (1.69)	2.05 (1.71)	–
LONG	30	26–40	4.45 (3.36)	5.61 (4.30)	–	–	3.20 (2.44)	3.43 (2.58)	–
CYCLIC	28	10–25	2.64 (2.26)	3.96 (3.56)	3.84*	4.62 (4.29)	1.96 (1.68)	2.50 (2.14)	3.71*
CYCLIC	34	10–40	2.68 (2.09)	4.18 (3.81)	4.23*	–	1.99 (1.55)	2.71 (2.37)	4.02*
ALL	106	9–40	3.15 (2.48)	4.04 (3.31)	–	–	2.32 (1.92)	2.65 (2.17)	–

B-RMSD, backbone root mean square deviation; N, number of peptides evaluated in each class; L, peptide length. APPTEST 1st model is that with the lowest XPLOR-NIH energy. PEP-FOLD 1st model is the one with the lowest sOPEP energy value. PEP-FOLD 3.5 was used to predict short and long peptide structures; PEP-FOLD 2.0 was used to predict cyclic peptide structures. PEPstrMOD only returns a single structure. PepLook results are taken from Beaufays *et al.* [4]

#### Short peptides

A total of 42 short (9-25 aa) peptides are predicted; the selected peptides are those originally evaluated for the PEPstr program [23]. Overall, APPTEST performs best, with its mean B-RMSD values being lower than the corresponding PEP-FOLD and PEPstrMOD values. Closer inspection reveals that of the best full structure predictions, APPTEST scores best on 27 of the 42 structures and PEP-FOLD 3.5 scores best on the remaining 15 structures. The results for just the RCs are similar, with APPTEST scoring best on 26 structures and PEP-FOLD scoring best on 16 structures.

As the best structure is one of a hundred structures, the 1st structure, i.e., the one with the lowest energy, should also be as close as possible to the experimental structure. Of the 42 structures investigated, APPTEST predicts the most-native structure for 25 of the peptides, PEP-FOLD 3.5 for 14 peptides and PEPstrMOD for the remaining 3. The RC results are similar, with APPTEST, PEP-FOLD and PEPstrMOD scoring 25, 15 and 2, respectively.

#### Long peptides

A total of 30 long (26-40 aa) peptides are predicted; the selected peptides are those originally evaluated for the PEP-FOLD program [32]. Prediction of peptide structures is increasingly challenging as the sequence length increases, as evidenced by APPTEST’s mean best full structure B-RMSD being 3.20 Å for long peptides, compared to 1.96 Å for short peptides. Nonetheless, APPTEST still outperforms PEP-FOLD 3.5 in this task, which has a mean B-RMSD of 3.43 Å. PEPstrMOD, meanwhile, does not facilitate predictions of peptides with a sequence length greater than 25 amino acids.

APPTEST and PEP-FOLD 3.5 return the most-native predictions for 16 and 14 of the 30 peptide structures included in this benchmark when the full structure B-RMSD are considered; this rises to 19 and 11 when only the structures’ RCs are considered. When the comparison is restricted to consider only the lowest energy structure returned by each method, APPTEST and PEP-FOLD 3.5 return the most-native structure for 20 and 10 of the peptides, respectively, for both the full structures and RC regions.

#### Cyclic peptides

The structures of cyclic peptides, such as those possessing disulfide bonds, have traditionally been challenging to predict. Neither PEPstr [23], the Gps algorithm [24] nor PEP-FOLD 1.0 [29] facilitated the prediction of cyclic peptide structures.

The 34 cyclic peptides in this benchmark set are between 10 and 30 amino acids in length and were originally used to evaluate the PEP-FOLD 2.0 and PepLook programs [28, 31]. As PEPstrMOD only facilitates predictions of peptides up to 25 amino acids in length, mean RMSD values are reported for the subset of 28 peptides with a sequence length of 10–25 amino acids, as well as for the entire set of 34 peptides. As the PepLook web server is no longer available, the B-RMSD values that follow are taken from the PepLook article [28]. Unfortunately, the values presented are for only the full structure or only the RC, and so the comparison with PepLook is consequently not fully comprehensive. As per the instructions on the PEP-FOLD web server, PEP-FOLD 2.0 is used instead of the newer PEP-FOLD 3.5 for the prediction of cyclic peptide structures.

Comparing the performance of the four methods on the cyclic dataset, it is clear that APPTEST outperforms the existing methods, with a mean best full structure B-RMSD value of 1.96 Å, compared to the 2.50 Å, 3.71Å and 4.62 Å of PEP-FOLD, PepLook and PEPstrMOD, respectively. This is further exemplified by inspecting predictions of individual structures: APPTEST returns the most native structure for 26 of the 34 peptides in the benchmark, and PEP-FOLD 2.0 returns the most native structure for the remaining 8 peptides. Considering only the RC regions, APPTEST returns the most accurate structure for 30 of the 34 peptides, with PEP-FOLD 2.0 scoring better on the remaining 4 peptides. Restricting the comparison to only consider the prime structure returned by each method, APPTEST achieves the best performance on 23 of the structures, with the remaining 6, 4 and 1 structures being best predicted by PEP-FOLD 2.0, PepLook and PEPstrMOD, respectively. The results when considering only the RC region are similar, with APPTEST, PEP-FOLD 2.0 and PepLook achieving the best performance for 25, 7 and 2 of the 34 structures, respectively.

**Key Points**

-
$\bullet $ A novel protocol was developed for the prediction of peptide tertiary structures from their primary structure.
-
$\bullet $ An artificial neural network model was constructed and trained for the prediction of structure distance and torsion angle restraints.
-
$\bullet $ Traditional NMR structure calculation software was used to obtain final ensembles of model structures from the generated restraints.
-
$\bullet $ APPTEST was evaluated using a redundancy-reduced external test set of 356 peptide sequences.
-
$\bullet $ APPTEST outperforms the current best-in-class methods for peptide tertiary structure prediction.

## Conclusion

To conclude, knowledge of a peptide’s tertiary structure is an important component in thoroughly understanding its biological activity. Elucidating tertiary structure is a non-trivial, time-intensive task that requires specialized equipment. This work briefly reviewed the history of peptide structure prediction and compared the most recent methods for peptide tertiary structure prediction. Furthermore, in order to facilitate a more accurate *de novo* prediction of peptide tertiary structure, the authors have developed a computational protocol that combines the predictive power of neural networks with existing structural biology software programs XPLOR-NIH and CYANA. Neural networks were trained on experimentally obtained model structures from the PDB to predict structural restraints, which are used in restrained molecular dynamics simulations to yield a final ensemble of structures. A test set of 356 peptides was used to evaluate the protocol’s performance, with mean B-RMSD values of 1.91 and 2.10 Å achieved when using XPLOR-NIH and CYANA, respectively. Additionally, the model’s performance was benchmarked against existing methods, PEP-FOLD, PEPstrMOD and PepLook, on sets of short, long and cyclic peptides. APPTEST achieved state-of-the-art performance on all three peptide sets, demonstrating that the combination of a neural network architecture with traditional structure determination methods is capable of accurately predicting peptide tertiary structure. Nonetheless, the proposed protocol possesses limitations: the sequence length is limited to 40 residues, which must be natural amino acids. These limitations may be overcome in future iterations of this work. This work extends a suite of existing *in silico* methods developed by the authors, which includes methods for the prediction of peptide hemolytic, anticancer, antiviral and anti-coronavirus activities [39–41]. The authors trust that the results of this work, in combination with the aforementioned classifiers, will facilitate improved *in silico* design of novel peptide-based therapeutics, thereby lowering reliance on specialized equipment and reducing the time and cost required for the design phase, helping to drive medicinal chemistry into an unprecedented revolution.

## Availability

### Web server

APPTEST is available as an easy to use web server online at https://research.timmons.eu/apptest, for the benefit of the wider scientific community. The web server is capable of predicting peptides’ tertiary structure based on the primary sequence and cyclic restraints. Input peptide sequences are restricted to the 20 natural amino acids; support for the prediction of peptides containing non-natural amino acids is not currently available. Users may choose to only predict the distance and angle restraints or to also conduct simulated annealing to produce an ensemble of model structures. Simulated annealing can be carried out only using XPLOR-NIH on the web server, as the authors’ CYANA license does not extend to usage in a web server context.

### Standalone

APPTEST is also available as a standalone executable program for Linux. This program has been tested to work with Ubuntu 20.04 LTS and Debian 10. The program can be downloaded from https://research.timmons.eu/apptest or alternatively can be requested from the authors. The standalone program is recommended for users who intend to carry out a large number of structure predictions.

## Supplementary Material

supplementary_bib_bbab308

## Notes

**Patrick Brendan Timmons** is a PhD researcher at the Conway Institute, University College Dublin. His research focuses on the computational modelling of peptide structures, and the application of deep learning to the study of therapeutic peptide structure and function.

**Chandralal M. Hewage** is an Associate Professor at the Conway Institute, University College Dublin. His research focuses on the use of NMR spectroscopy to study the structures of bioactive peptides.
