# Supplementary material for: APPTEST is a novel protocol for the automatic prediction of peptide tertiary structures
Source: Brief Bioinform. 2021 Aug 14;22(6):bbab308. doi: 10.1093/bib/bbab308 (PMC8575040; doi:10.1093/bib/bbab308)
Supplement: supplementary_bib_bbab308 [file supplementary_bib_bbab308.pdf]

APPTEST is a novel protocol for the automatic prediction of peptide tertiary structures - Supplementary Information

Patrick Brendan Timmons[1*],
Chandralal M. Hewage[1]

[1]UCD School of Biomolecular and Biomedical Science, UCD Centre for Synthesis and Chemical Biology, UCD Conway Institute, University College Dublin, Dublin 4, Ireland
[*]patrick.timmons@ucdconnect.ie

Table 1: APPTEST performances on the APPTEST independent test set, using XPLOR-NIH for torsion angle dynamics and simulated annealing. B-RMSD values are given for the best-predicted model, and the model with the lowest energy. Numbers in brackets are B-RMSD values for the peptides' rigid cores only.

PDB	Sequence	Length	Rigid Core Region	Prime	Best
1a13	INWKGIAAMAKKLL	14	2-14	2.26 (2.05)	2.02 (1.89)
1a1p	ICVVQDWGHHRCT	13	1-13	2.87 (2.87)	1.64 (1.64)
1a93	CGGVQAEEQKLISEEDLLRKRREQLKHKLEQL	32	1-32	0.89 (0.89)	0.79 (0.79)
1ard	RSFVCEVCTRAFARQEHLKRHYRSHTNEK	29	1-24,27	1.64 (1.3)	1.64 (1.3)
1b03	RKSIRIQRGPGRAFVTIG	18	1-18	5.05 (5.05)	2.85 (2.85)
1b1v	ENFNGGCLAGYMRTADGRCKPTF	23	7,9,10-14,17-23	3.92 (2.23)	3.02 (1.89)
1b45	GRCCHPACGKYYSC	14	2-9,13-14	1.75 (1.21)	1.36 (1.13)
1bde	YGDTWAGVEAIIRILQQLLFIHFRIGCRHSRIG	33	4-29	2.01 (0.91)	1.42 (0.68)
1by0	RKKLEELERDLRKLKKKIKKLEEDNPW	27	2-4,6-15	1.68 (0.67)	1.42 (0.64)
1c6w	GDCLPHLKLCKENKDCCSKKCKRRGTNIEKRCR	33	2-25,27-33	2.68 (2.65)	2.44 (2.38)
1c98	GNLWATGHFM	10	1-10	2.84 (2.84)	2.3 (2.3)
1cb3	IDYWLAHKALA	11	2-5	1.88 (0.57)	1.29 (0.35)
1d1f	ARHYKNLIERQRY	13	1-12	1.02 (1)	0.9 (0.88)
1d6x	VRRFPWWWPFLRR	13	1-12	2.53 (2.5)	2.34 (2.16)
1dfw	FPIPLPYCWLCRALIKRIQAMIPKG	25	6-20,22-24	4.94 (3.09)	3.62 (2.39)
1dkc	AGCIKNGGRCNASAGPPYCCSSYCFQIAGQSYGVCKNR	38	4-5,7-10,19-37	2.36 (1.87)	2.19 (1.85)
1dn3	QAPAYKKAAKKLAES	15	1-15	1.1 (1.1)	1.02 (1.02)
1dpu	RIQRNKAAALLRLAAR	16	4-14	1.56 (0.41)	1.43 (0.28)
1dsj	YGDTWAGVEAIIRILQQLLFIHFRIG	26	2-26	1.75 (1.64)	1.13 (1)
1du1	TSAQKAKAEERKRRKMSRGL	20	1-20	4.94 (4.94)	4.16 (4.16)
1dxz	PTDSGEKMTLSISVLLSLTVFLLVIVELIPST	32	5,11,12,15,16-32	1.99 (0.92)	1.16 (0.71)
1e0m	SMGLPPGWDEYKTHNGKTYYYNHNTKTSTWTDPRMSS	37	3-35	2.75 (2.32)	2.07 (1.79)
1e0n	PGWEIIHENGRPLYYNAEQKTKLHYPP	27	1-25	6.85 (6.42)	3.73 (3.75)
1e0q	MQIFVKTLDGKTITLEV	17	1-17	3.77 (3.77)	2.79 (2.79)
1e75	GCCSDPLCAWRC	12	1-12	1.97 (1.97)	1.34 (1.34)
1egs	TKSAGGIVL	9	1-9	6.52 (6.52)	3.65 (3.65)
1etl	CELCCNPGCAGC	12	1-12	2.05 (2.05)	1.33 (1.33)
1f2s	RICPRIWMECKRDSDCMAECICVMGHCG	28	1-28	2.09 (2.09)	1.52 (1.52)
1fge	CEAPEGYILDDGFICTDIDE	20	1-20	3.1 (3.1)	2.57 (2.57)
1fmh	EVAQLEKEVAQAEAENYQLEQEVAQLEHECG	31	1-31	0.59 (0.59)	0.41 (0.41)
1g0v	TDQQKVSEIFQSSKEKLQGDAMVVSDAFK	29	1-29	0.89 (0.89)	0.76 (0.76)
1g3f	AVPIAQKSE	9	1-9	2.17 (2.17)	1.53 (1.53)
1g89	ILPWKWPWWPWRR	13	2-12	2.55 (2.39)	1.82 (1.64)
1gjf	RAGPLQWLAEKYQG	14	1-14	1.86 (1.86)	0.96 (0.96)
1hc9	WRYYESSLLPYPD	13	1-13	4.13 (4.13)	2.72 (2.72)
1hly	TVIDVKCTSPKQCLPPCKAQFGIRAGAKCMNGKCKCYPH	39	1-38	1.54 (1.45)	1.44 (1.38)
1hu5	KNLRRIIRKIIHIIKKYG	18	2-18	1.96 (1.79)	1.61 (1.48)
1hv2	TLKERCLQVVRSLVK	15	1-15	0.63 (0.63)	0.36 (0.36)
1id6	SVQARWEAAFDLDLY	15	1-14	5.54 (5.59)	4.96 (5.02)
1ien	FNWRCCLIPACRRNHKKFC	19	1-19	2.35 (2.35)	1.56 (1.56)
1im7	IWGCSGKLICTTA	13	1-13	3.15 (3.15)	2.08 (2.08)
1ir3	GDYMNM	6	1-6	1.99 (1.99)	1.3 (1.3)
1ixu	FATMRYPSDSDE	12	1-12	4.52 (4.52)	3.64 (3.64)
1j4m	RGKWTYNGITYEGR	14	1-14	3.17 (3.17)	1.93 (1.93)
1j5b	DVASDAKAAAELVAANAKAAAELVAANAKAAAEAVAR	37	1-37	0.86 (0.86)	0.86 (0.86)
1j5j	RPTDIKCSESYQCFPVCKSRFGKTNGRCVNGFCDCF	36	1-36	1.56 (1.56)	1.39 (1.39)
1jbu	EEWEVLCWTWETCER	15	1-15	3.61 (3.61)	2.96 (2.96)
1jgd	RRLLRGHNQY	10	1-10	2.39 (2.39)	1.59 (1.59)
1jq9	FLSYK	5	1-5	1.4 (1.4)	0.93 (0.93)
1k9r	PLPPY	5	1-5	0.64 (0.64)	0.39 (0.39)
1ka7	RKSLTIYAQVQK	12	1-12	3.24 (3.24)	2.12 (2.12)
1kal	SWPVCTRNGLPVCGETCVGGTCNTPGCTC	29	1-22,24-29	3.07 (3.1)	2.2 (2.17)
1kat	GGNECDIARMWEWECFERL	19	5-19	4.21 (3.83)	2.57 (2.28)
1kcn	ALCPAVCYVGGKALCPDVCYV	21	2-9,14-21	2.72 (2.48)	2.41 (2.11)
1kio	EVTCEPGTTFKDKCNTCRCGSDGKSAACTRMACPQ	35	2,4,5-30,33	2.4 (2.23)	2.04 (1.82)
1kl5	HPQFE	5	1-5	2.91 (2.91)	0.89 (0.89)
1kvg	SCHFGPLGWVCK	12	1-12	1.42 (1.42)	1.42 (1.42)
1kwd	PCSICSNNPTCWAICK	16	2-15	2.56 (2.29)	1.93 (1.53)
1kz5	RQIKIWFRKWKK	12	1-12	3.37 (3.37)	2.35 (2.35)
1l2i	KHKILHRLLQ	10	1-10	2.31 (2.31)	1.65 (1.65)
1l3q	FPGKNVNCTSGE	12	1-12	5.98 (5.98)	3.8 (3.8)
1lb7	RNCFESVAALRRCMYG	16	2-16	2.53 (2.53)	1.91 (1.81)
1m02	HPLKQYWWRPSI	12	2-11	4.23 (3.13)	2.39 (1.86)
1m7l	GLPDVASLRQQVEALQGQVQHLQAAFSQYKKVELFPNGGI	40	6-14,16-24,26-27,29-35	2.92 (1.79)	2.14 (1.33)
1m8o	KVGFFKRNRPPLEEDDEEGE	20	1-17	5.69 (4.87)	3.83 (3.26)
1mb6	ECLEIFKACNPSNDQCCKSSKLVCSRKTRWCKYQI	35	1-35	1.59 (1.59)	1.47 (1.47)
1mdi	FRFRYVCEGPSHG	13	1-13	4.98 (4.98)	2.83 (2.83)
1mf4	VAFRS	5	1-5	1.81 (1.81)	1.33 (1.33)
1mt7	VSQNYPIV	8	1-8	1.48 (1.48)	1.27 (1.27)
1mxe	IKKNFAKSKWKQAFNATAVVRHMRK	25	1-25	1.37 (1.37)	1.37 (1.37)
1myu	DVPKSDQFVGLM	12	2-12	1.73 (1.42)	1.22 (1.19)
1n0a	CTWEPDGKLTC	11	1-11	2.38 (2.38)	0.9 (0.9)
1n0c	CHWEGNKLVC	10	1-10	0.74 (0.74)	0.59 (0.59)
1n6t	HKTDSFVGLM	10	1-10	0.56 (0.56)	0.5 (0.5)
1niz	KRIHIGPGRAFYTT	14	1-14	2.32 (2.32)	1.74 (1.74)
1nkf	DKDGDGYISAAEAAAQ	16	1-16	5.41 (5.41)	4.13 (4.13)
1o06	EEDPDLKAAIQESLREAEEA	20	1-20	1.65 (1.65)	0.93 (0.93)
1oby	NEFYA	5	1-5	0.64 (0.64)	0.4 (0.4)

1odp	YSDELRQRLAARLEALKENG	20	1-20	1.83 (1.83)	1.34 (1.34)
1oig	RPECVLNSDCPSNQACVNQKCRDP	24	1-11,14-23	2.98 (2.66)	2.8 (2.46)
1ou8	GAANDENY	8	1-8	2.8 (2.8)	1.76 (1.76)
1ox1	SCTRSIPPQC	10	1-10	2.16 (2.16)	1.48 (1.48)
1p0j	AKKVFKRLEKLFSKIQNWK	19	2-19	1.58 (1.3)	1.27 (1.11)
1p0l	AKKVFKRLEKLFSKIWNDK	19	3-19	1.66 (0.89)	1.27 (0.81)
1p5k	AKKVFKRLEKSFSKIQNDK	19	2-18	1.29 (1.03)	1.21 (0.72)
1p7w	PAPFASA	7	1-7	1.99 (1.99)	1.41 (1.41)
1pef	EQLLKALEFLLKELLEKL	18	1-18	0.6 (0.6)	0.6 (0.6)
1pon	VTEEDIEDLMKDSDKNNDGRIDFDEFLKMMEGVQ	34	3,6,7-12,20-30	4.06 (3.76)	3.19 (3.11)
1q0w	YPEDEEELIRKAIELSLKESRNSA	24	3-21	2.17 (0.58)	1.37 (0.5)
1qcm	GSNKGAIIGLM	11	3-11	2.95 (3.1)	2.72 (2.58)
1qfa	LRHYLNLLTRQRY	13	1-13	1.01 (1.01)	0.9 (0.9)
1qfd	CIPKWNRCGPKMDGVPCCEPYTCTSDYYGNCS	32	1-32	2.79 (2.79)	2.26 (2.26)
1qg9	NVEYTFTGIYTFESLIKILAR	21	6,9,10-18	1.82 (0.85)	1.24 (0.56)
1qk6	ACKGVFDACTPGKNECCPNRVCSDKHKWCKWKL	33	1-13,16-33	1.34 (1.2)	1.24 (1.15)
1r8t	RCCHPQCGAAYSCRK	15	1-14	1.89 (1.79)	1.68 (1.56)
1rff	KLNYYD	6	1-6	3.87 (3.87)	2.23 (2.23)
1rgj	FRYYESSLEPWDD	13	1-13	5.83 (5.83)	3.54 (3.54)
1rji	TPYPVNCKTDRDCVMCGLGISCKNGYCQGCT	31	1-31	2.45 (2.45)	2.36 (2.36)
1rpv	TRQARRNRARRWRARQR	17	4-16	0.86 (0.35)	0.63 (0.28)
1rst	AWRHPQFGG	9	1-9	4.18 (4.18)	3.35 (3.35)
1s7q	AVYNFATM	8	1-8	0.84 (0.84)	0.84 (0.84)
1skk	KKWWKF	6	1-6	4.17 (4.17)	2.03 (2.03)
1sle	CHPQGPPC	8	1-8	1.7 (1.7)	1.32 (1.32)
1slg	SHPQNT	6	1-6	3.43 (3.43)	1.57 (1.57)
1sol	KHVVPNEVVVQRLFQVKGRR	20	1-20	4.14 (4.14)	2.56 (2.56)
1str	CHPQNT	6	1-6	1.94 (1.94)	1.02 (1.02)
1ter	ALCNCNRIIIPHMCWKKCGKK	21	2-21	4.33 (3.77)	4.33 (3.77)
1tjb	YIDTNNDGWYEGDELLA	17	1-17	6.03 (6.03)	3.78 (3.78)
1tn6	DDPTASACNIQ	11	1-11	3.11 (3.11)	2.04 (2.04)
1u0i	EIAALEKEIAALEKEIAALEK	21	1-19	0.66 (0.4)	0.46 (0.4)
1use	SSDYSDLQRVKQELLEEVKKELQKVKEEIIEAFVQELRKR	40	1-40	3.05 (3.05)	2.1 (2.1)
1v5a	RCLPSGKACAGVTQKIPCCGSCVRGKCS	28	1-9,12,14,15-28	1.81 (1.76)	1.74 (1.68)
1v6r	CSCSSLMDKECVYFCHLDIIW	21	1-21	4.99 (4.99)	3.87 (3.87)
1vm4	GLFDIVKKLVSDF	13	1-13	0.65 (0.65)	0.54 (0.54)
1wlp	GPLGSKQPPSNPPPRPPAEARKKPS	25	1-25	4.52 (4.52)	3.31 (3.31)
1wm8	VGCEECPMHCKGKNANPTCDDGVCNCNV	28	1-28	2.3 (2.3)	2.21 (2.21)
1wr4	GSPGLPSGWEERKDAKGRTYYVNHNNRTTTWTRPIM	36	5-6,8-14,16,18,19-35	7.36 (6.83)	2.22 (1.63)
1ws4	QSGISQTVIVGPWGAKSA	18	1-18	4.2 (4.2)	1.73 (1.73)
1x7k	RRWCFRVCYRGRFCYRKCR	19	4,8,9-10,13-18	3.63 (2.38)	2.17 (1.85)
1xc0	GFFALIPKIISSPLFKTLLSAVGSALSSSGGQE	33	1-29	4.09 (3.65)	3.14 (2.9)
1xha	TTYADFIASGRTGRRNAIHD	20	1-20	4.06 (4.06)	2.49 (2.49)
1y58	CRRWQWRMKKLGC	13	1-13	3.72 (3.72)	2.83 (2.83)
1yiu	GAMGPLPPGWEKRTDSNGRVYFVNHNTRITQWEDPRS	37	5-35	2.11 (1.71)	2 (1.58)
1ymz	MPLPPGWERRTDVEGKVYYFNVRTLTTTWERPTIILE	37	2-13,15-33	2.37 (1.65)	2.1 (1.65)
1yp0	HPTILYTLLSPG	12	1-12	3.71 (3.71)	2.34 (2.34)
1ytv	NSSSN	5	1-5	2.62 (2.62)	1.75 (1.75)
1zmp	ATCYCRTGRCATRESLSGVCEISGRLYRLCCR	32	1-32	1.64 (1.64)	1.61 (1.61)
1znf	YKCGLCERSFVEKSALSRHQRVHKN	25	1-24	1.79 (1.65)	1.71 (1.65)
1zub	EEGIWA	6	1-6	1.93 (1.93)	1.1 (1.1)
1zuz	RRRWKLDFSIVSLCNHLTR	19	1-19	2.02 (2.02)	1.07 (1.07)
1zvb	ALNTLVKQLSSNFGAISSVLNDILSRLDKVEAEV	34	1-34	4.01 (4.01)	3.73 (3.73)
2a2v	ECRKMFGGCSVDSDCCAHLGCKPTLKYCAWDGTF	34	1-32	1.72 (1.31)	1.46 (1.31)
2a40	SDARSDLLSAIRQGFQLRRVEE	22	1-22	4.68 (4.68)	4.29 (4.29)
2ab3	MVYVCHFENCGRSFNDRRKLNRHKKIHTG	29	1-29	2.61 (2.61)	2.41 (2.41)
2ajj	SGNYVLDLIYSLHKQINRGLKKIVLGWA	28	4-27	2.09 (1.31)	1.69 (0.94)
2ap7	IIGPVLGLVGSALGGLLKKI	20	3-20	1.25 (0.75)	0.9 (0.69)
2axf	APQPAPENAY	10	1-10	2.7 (2.7)	1.68 (1.68)
2beq	SQIQESLTTTSTALGKLQDVVNQNAQALNTLVKQLS	36	1-36	1.64 (1.64)	1.13 (1.13)
2bta	MEELQDDYEDMMEEN	15	1-15	4.53 (4.53)	3.08 (3.08)
2cik	KPIVVLHGY	9	1-9	2.17 (2.17)	1.41 (1.41)
2czy	APQLIMLANVALTGE	15	1-14	1.59 (1.04)	1.22 (0.99)
2d2p	HSDGIFTDSYSRYRKQMAVKKYLAAVLGKRYKQRVKNK	38	7-8,11,14,15,17,18-35,37	2.08 (1.23)	2.08 (1.12)
2dci	GLFGAIAGFIENGAEGMIDG	20	1-11,13	2.69 (1.54)	2.19 (1.39)
2ddl	AFCNLRMCQLSCRKSLGCLLGKCIGDKCKCYGC	33	2-13,15-18,20-21,23-24,28-30	1.63 (1.32)	1.6 (1.2)
2drn	DLIEEAASRIVDAVIEQVKAAGAY	24	1-20	1.01 (0.67)	0.83 (0.57)
2efz	VCCPFGGCHELCYCCD	16	1-16	5.03 (5.03)	1.75 (1.75)
2f3a	RLFDKIRQVIRKF	13	1-13	1.03 (1.03)	0.87 (0.87)
2fvj	HKLVQLLTTT	10	1-10	0.89 (0.89)	0.89 (0.89)
2g1t	EIFGEFE	7	1-7	2.41 (2.41)	1.95 (1.95)
2gpo	SLLLHLLKSQ	10	1-10	1.1 (1.1)	0.99 (0.99)
2gtz	ALGIGILTV	9	1-9	1.62 (1.62)	1.29 (1.29)
2hjl	KAFNPEIIPMF	11	1-11	1.73 (1.73)	1.23 (1.23)
2hqw	KKKATFRAITSTLASSFKRRRS	22	1-22	2.05 (2.05)	1.62 (1.62)
2ht9	LGTENLYFQSME	12	1-12	2.25 (2.25)	1.62 (1.62)
2hug	APPGTARRKRKADS	14	11,13	4.16 (1.02)	2.72 (0.46)
2hy6	VKQLADAVEELASANYHLANAVARLAKAVGER	32	1-32	0.61 (0.61)	0.61 (0.61)
2i1e	VKKFPWWWPFLKK	13	2-13	3.1 (2.62)	2.2 (1.92)
2i1g	VRRYPWWWPYLRR	13	2-13	3.23 (3.1)	2.17 (2.01)
2i1i	VRRFPWWWAFLRR	13	1-13	3.94 (3.94)	2.94 (2.94)
2i28	GCCSTPPCAVLYC	13	1-13	2.14 (2.14)	1.65 (1.65)
2i5o	AEDQVPCEKCGSLVPVWDMPEHMDYHFALELQKS	34	3-33	4.57 (3.67)	3.89 (3.39)
2i94	MDFGSLETVVANSAFI	16	2,4,5-16	1.3 (1.09)	1.3 (0.84)
2ifi	GCCSDARCAWRC	12	1-12	3.35 (3.35)	2.25 (2.25)
2ivz	GASDGSGWSSENNPWG	16	1-16	5.58 (5.58)	4.14 (4.14)
2j10	EYFFLKIRGRERFEMFRELNEALELKDAQAG	31	1-31	2.09 (2.09)	1.72 (1.72)
2j7y	QSLINLLAD	9	1-9	2.01 (2.01)	1.63 (1.63)
2jam	GVSKFA	6	1-6	1.05 (1.05)	0.95 (0.95)

2jnw	KIIDTGGGFIL	11	1-11	4 (4)	2.68 (2.68)
2jqu	GGSLYSFGL	9	1-9	3.81 (3.81)	3.04 (3.04)
2jtt	SEGLMNVLKKIYEDGDDDMKRTINKAWVESR	31	4-14,18-30	2.87 (2.29)	2.55 (2.04)
2jtw	KKSHTASYLRLWALSLAHAQLSSKK	25	2-25	3.21 (3.25)	2.26 (2.21)
2juq	GCCSDVRCRYRCR	13	1-5,7-8,12	1.44 (1.36)	1.4 (1.25)
2jxf	QTNWQKLEVFWAKHMWNFISGIQYLAGLST	30	3-26,28	1.1 (0.59)	1.1 (0.59)
2k1p	GSSANDWQCKTCSNVNWARRSECNMCNTPKYAK	33	5-32	4.26 (3.66)	3.76 (2.98)
2k4u	VGINVKCKHSRQCLKPCKDAGMRFGKCTNGKCHCTPK	37	1-37	1.35 (1.35)	1.3 (1.3)
2k76	PFPPTPPGEEAPVEDLIRFYNDLQQYLNVV	30	2-28,30	9.91 (8.97)	5.95 (5.53)
2kgu	GYCAEKGIRCDDIHCCTGLKCKCNASGYNCVCRKK	35	2-35	2.12 (2.06)	2.02 (2)
2khf	GAWKNFWSSLRKGFYDGEAGRAIRR	25	2-24	3.1 (2.77)	2.94 (2.7)
2ki0	GSGQVRTIWVGGTPEELKKLKEEAKKANIRVTFWGD	36	8-10,13-25,30-34	11.92 (8.3)	8.38 (6.92)
2km9	CKGTGKSCSRIAYNCCTGSCRSGKC	25	1-25	1.46 (1.46)	1.35 (1.35)
2krb	DEDVKDNWDDD	11	1,3,4-9,11	4.12 (4.32)	2.62 (2.58)
2kus	SGPNGQCGPGWGGCRGGLCCSQYGYCGSGPKYCAH	35	1-15,17-34	3.55 (3.47)	3.26 (3.21)
2kux	GTPCGESCVYIPCISGVIGCSCTDKVCYLN	30	1-30	2.3 (2.3)	1.66 (1.66)
2kxc	HIPPAPNWPAPTPPVQN	17	1-16	2.94 (2.99)	1.99 (1.9)
2kym	GKFIPSRPAPKPPSSA	16	2-8,11,14	2.7 (1.84)	2 (1.66)
2l07	DCKRKVYPNGSISDYCEY	18	2-17	2.56 (2.52)	2.23 (2.09)
2l0g	GHMFPSDIDPQVFYELPEAVQKELLAEWKRTG	32	4-5,7-32	3.73 (2.24)	2.27 (1.33)
2l5g	KKEMEERMSLEETKEQILKLEEKLLALQEEKHQLFLQL	38	5-8 ,3,9,11,12-17,19-35	2.45 (2.08)	2.45 (1.87)
2l7c	QPFTKGAYYIGKMVWSKGY	19	3-5,8-16	1.75 (1.21)	1.75 (1.03)
2lbg	KHMAGAAAAGAVVGGLGGYMLGSAMSR	27	1-27	1.61 (1.61)	1.08 (1.08)
2lcm	KDINTIKSLRVLRVLRPLKTIKRLPKLK	28	2-26	3.17 (1.93)	2.22 (1.54)
2lix	ACVTHEDCTLLCYDTIGTCVDGKCKCM	27	1-27	5.5 (5.5)	3.23 (3.23)
2lo9	VGERCCKNGKRGCGRWCRDHSRCC	24	1-24	2.31 (2.31)	2.31 (2.31)
2lqy	HGLASTLTRWAHYNALIRAF	20	1-18	2.29 (1.43)	1.75 (1.1)
2lz5	GCCSDPPCRNKHPDLC	16	1-16	1.19 (1.19)	1.05 (1.05)
2m6a	ADDRCERMCQRYHDRREKKQCMKGCRYG	28	3-27	2 (1.29)	1.71 (1.29)
2m7t	GCPQGRGDWAPTSCSQDSDCLAGCVCGPNGFCG	33	1-33	3.2 (3.2)	2.49 (2.49)
2m8m	NEQELLELDKWASLWNWFNITNWLWYIK	28	5-10,14-15,17-24	1.64 (0.83)	1.5 (0.78)
2mau	CAQKGEYCSVYLQCCDPYHCTQPVIGGICA	30	1-30	2.58 (2.58)	2.35 (2.35)
2mg1	KKKLFIMIVGGLVGLRIVFAVLSIKKK	27	1-27	1.01 (1.01)	0.93 (0.93)
2mjq	GLLKRIKTLL	10	1-10	0.42 (0.42)	0.33 (0.33)
2mjr	GLLKWIKTLL	10	1-10	0.47 (0.47)	0.23 (0.23)
2mjs	GLLKKIKWLL	10	1-10	0.42 (0.42)	0.4 (0.4)
2ml7	CKSGGAWCGFDPHGCCGNCGCLVGFCYGTGC	31	1-31	2.44 (2.44)	2.29 (2.29)
2mlq	EKFRRYLSVFFRKHIT	16	3-14	2.25 (1.48)	1.82 (1.35)
2mm6	CRPYGYRCDGVINQCCDPYHCTPPLIGICL	30	1-30	2.1 (2.1)	1.85 (1.85)
2moc	TGKASQFFGLM	11	1-11	1.69 (1.69)	0.8 (0.8)
2mso	SCNNSCQSHSDCASHCICTFRGCGAVNGLP	30	1-26,29-30	2.75 (2.71)	2.35 (2.35)
2mtw	YTNQNINISQERDLQKHGFH	20	1-20	3.81 (3.81)	3.05 (3.05)
2mu9	KMNMLKENVDYIQKNQNLFK	20	1-16,18	1.32 (1.2)	1.11 (1)
2mw7	DGECGDKDEPCCGRPDGAKVCNDPWVCILTSSRCENP	37	2-15,17-37	4.57 (4.43)	3.63 (3.45)
2mxp	QMQPLCFNCPICDKIFPATEKQIFEDHVFCHSL	33	5-33	6.36 (5.37)	3.64 (3.69)
2n5r	VRRFDLLKRILK	12	1-12	1.49 (1.49)	1.21 (1.21)
2n6m	GIFSKLAGKKIKNLLISGLKG	21	1-21	1.98 (1.98)	1.82 (1.82)
2n6o	ECRYLFGGCSSTSDCCKHLSCRSDWKYCAWDGTFS	35	1-35	2.1 (2.1)	1.94 (1.94)
2n8s	GSPSPLPPGWEERQDVLGRTYYVNHESRTTQWKRPSPED	39	32-33,7-10,18-19,21-24,29-31	3.26 (1.14)	3.09 (1.14)
2nlq	DHYNCVSSGGQCLYSACPIFTKIQGTCYRGAAKCCKG	37	1-37	3.7 (3.7)	3.2 (3.2)
2nr1	DALTLSSAMWFSWGVLLNSGIGE	23	1-22	0.79 (0.71)	0.79 (0.69)
2nx7	AQNPCSLQQPGCSSACAPACRLSCCSLG	28	2-28	5.94 (6.04)	3.52 (3.53)
2nz3	ATCDLASIFNVNHALCAAHCIARRYRGGYCNSKAVCVCRN	40	3-13 ,10,14-17,19-28,36-40	3.64 (2.79)	3.08 (1.76)
2o02	GLLDALDLAS	10	1-10	3.86 (3.07)	2.23 (2.23)
2o02	GHGQGLLDALDLAS	14	1-14	3.86 (3.86)	3.24 (3.24)
2o8z	EAHKNRKLMEII	12	1-12	1.95 (1.95)	1.8 (1.8)
2oei	GSDLPAGWMRVQDTSGTYYWHIPTGTTQWEPPGRASP	37	1-37	3.98 (3.98)	3.06 (3.06)
2oi3	HSKYPLPPLPSL	12	1-12	2.69 (2.69)	2.01 (2.01)
2oq9	APMQAPVQAAPACMASCAPQCCGR	24	4,6,7-23	7.89 (6.41)	5.04 (4.23)
2os6	VENKVTDL	8	4-8	1.66 (1.17)	1.08 (0.69)
2ox2	RRWWFR	6	1-6	3.24 (3.24)	1.52 (1.52)
2p15	HKILHRLLQDS	11	1-11	1.1 (1.1)	0.63 (0.63)
2p7r	CPFVC	5	1-5	1.31 (1.31)	0.71 (0.71)
2pku	ESVKI	5	1-5	1.37 (1.37)	0.47 (0.47)
2pm4	DCYCRIPACIAGEKKYGTCIYQGKLWAFCC	30	1-30	1.32 (1.32)	1.28 (1.28)
2pta	TISCTNPKQCYPHCKKETGYPNAKCMNRKCKCFGR	35	2-30,32-34	1.43 (1.31)	1.39 (1.31)
2q0n	RRRRRSWYFDG	11	1-11	3.32 (3.32)	1.92 (1.92)
2qbw	PQPVDSWV	8	1-8	3.17 (3.17)	1.73 (1.73)
2qhr	VEQHHRRTDND	11	1-11	2.3 (2.3)	1.79 (1.79)
2qiy	LPLFINTTEAEFAAASVQRYELNMK	25	1-25	4.51 (4.51)	3.06 (3.06)
2r7g	PPTLHELYDL	10	1-10	3.12 (3.12)	2.2 (2.2)
2rol	PPVPNPDYEPIR	12	1-12	2.68 (2.68)	1.85 (1.85)
2rrh	HSDAVFTDNYTRLRKQMAVKKYLNSILNG	29	3-29	1.31 (1.01)	1.19 (0.69)
2run	LGKYEQYIKWPWYVWLGF	18	1-18	3.12 (3.12)	2.38 (2.38)
2v1v	RVCPRILLECKKDSDCLAECVCLEHGYCG	29	2-29	1.59 (1.59)	1.57 (1.52)
2wh9	EGECGGFWWKCGSGKPACCPKYVCSPKWGLCNFPMP	36	3-35	2.59 (2.43)	2.26 (2.04)
2xa7	DYQRLN	6	1-6	1.39 (1.39)	0.81 (0.81)
2y2a	KLVFFA	6	1-6	1.2 (1.2)	0.43 (0.43)
2y3k	MVGGVVIA	8	1-8	2.19 (2.19)	1.3 (1.3)
2y4v	KYKQSVRLISLCQRLS	16	1-16	0.8 (0.8)	0.28 (0.28)
2yle	SLYKIKPRHDSGIKAKISMKT	21	1-21	4.34 (4.34)	3.34 (3.34)
2yxr	ETFSKIRVKPEHVIGVTVAFVIIEAILTYGRF	32	1-32	4.69 (4.69)	3.06 (3.06)
3avc	SDKIDNLD	8	1-8	2.38 (2.38)	1.07 (1.07)
3bin	GTYFTHE	7	1-7	1.2 (1.2)	1.2 (1.2)
3bw9	CPSQEPMSIYVY	12	1-12	3.21 (3.21)	3.01 (3.01)
3c5j	QVIILNHPGQISA	13	1-13	5 (5)	3.58 (3.58)
3cbl	IYESL	5	1-5	1.2 (1.2)	0.67 (0.67)
3drk	SFANG	5	1-5	1.39 (1.39)	0.87 (0.87)
3e21	MDREMILADFQACTGIENIDEAITLLEQNNWDLVAAINGV	40	1-40	9.14 (9.14)	6.95 (6.95)

3e4h	CGETCTLGTCYTAGCSCSWPVCTRNGVPI	29	1-29	1.61 (1.61)	1.41 (1.41)
3ecb	RGPGRAFVTI	10	1-10	3.35 (3.35)	3.01 (3.01)
3iet	GTKPPL	6	1-6	4.35 (1.74)	0.88 (0.88)
3iet	GTKPPLEEL	9	1-9	4.35 (4.35)	3.01 (3.01)
3jrv	SSFFSDR	7	1-7	4.34 (4.34)	2.49 (2.49)
3kpe	FPSDEFDASISQVNEKINQSLAFIRKSDELLHNVN	35	1-35	2.83 (2.83)	2.46 (2.46)
3kpn	EEYLQAFTY	9	1-9	1.83 (1.83)	1.35 (1.35)
3kus	LNPNAKEFVPG	11	1-11	2.25 (2.25)	1.58 (1.58)
3kze	SRKEYYA	7	1-7	0.92 (0.92)	0.69 (0.69)
3ljm	EWEALEKKCAALESKLQALEKKLEALEHG	29	1-29	0.92 (0.92)	0.91 (0.91)
3ll8	EPIAIIITDTE	11	1-11	1.46 (1.46)	1.46 (1.46)
3ln5	HEEAVSVDRVL	11	1-11	2.66 (2.66)	1.9 (1.9)
3m53	SKSKDRKY	8	1-8	3.44 (3.44)	2 (2)
3m91	STAAGQERREKLTEETDDLLDEIDDVLEENA	31	1-31	0.92 (0.92)	0.89 (0.89)
3md4	GYMLGS	6	1-6	1.6 (1.6)	0.86 (0.86)
3mmg	ETVRFQSD	8	1-8	1.96 (1.96)	1.48 (1.48)
3mn5	PSPREQLMESIRKGKELKQI	20	1-20	3.69 (3.69)	2.97 (2.97)
3mrj	CINGMCWTV	9	1-9	1.34 (1.34)	0.9 (0.9)
3n3x	SDDDMG	6	1-6	1.8 (1.8)	1.1 (1.1)
3nvg	MIHFGN	6	1-6	1.63 (1.63)	1.01 (1.01)
3nvh	MIHFGND	7	1-7	1.53 (1.53)	1.28 (1.28)
3obx	PEATAPPEE	9	1-9	1.24 (1.24)	1.14 (1.14)
3oud	RVLFEAM	7	1-7	1.81 (1.81)	1.42 (1.42)
3p8n	KGSVVIVGRIILS	13	1-13	5.3 (5.3)	3.17 (3.17)
3pth	ELNPNAEVWGAPVLH	15	1-15	3.76 (3.76)	2.68 (2.68)
3q47	NPISDVD	7	1-7	2.95 (2.95)	1.48 (1.48)
3rc4	PSSTPC	6	1-6	1.27 (1.27)	1.01 (1.01)
3rf3	TRETIFEASKKVTNSLSNLISLIG	24	1-24	2.27 (2.27)	1.61 (1.61)
3sge	EEEDDDMGFGL	11	1-11	4.07 (4.07)	2.38 (2.38)
3sgs	GDVIEV	6	1-6	1.06 (1.06)	0.67 (0.67)
3skm	FLRGRAYVL	9	1-9	1.61 (1.61)	1.59 (1.59)
3thk	PPPVPP	6	1-6	0.53 (0.53)	0.36 (0.36)
3twg	EYKEEDQERRKRKKRS	16	1-16	4.23 (4.23)	3.34 (3.34)
3ujj	TRKSIRIGPGQAFYATG	17	1-17	4 (4)	2.96 (2.96)
3up3	NALLRYLLDKDDT	13	1-13	2.4 (2.4)	2.05 (2.05)
3v30	TLVSMPPLPGLDLKGS	16	1-16	3.08 (3.08)	2.48 (2.48)
3v43	ARTKQTA	7	1-7	2.67 (2.67)	2.03 (2.03)
3v5h	KVAEIVHFL	9	1-9	1.72 (1.72)	0.95 (0.95)
3vza	NAFSELDSADPRVMLRRIIQNQPQVDPLALQ	31	1-31	5.49 (5.49)	3.99 (3.99)
3zkt	ECCHRQLLCCLRFV	14	2-4,6-12	2.44 (1.48)	1.83 (1.37)
4a1v	DELVYLLDGPGYDPIHS	17	1-17	2.84 (2.84)	1.89 (1.89)
4axy	PPGPPGPTGPRGPPGPPG	18	1-18	1.86 (1.86)	0.75 (0.75)
4e0k	KDWSFY	6	1-6	1.15 (1.15)	0.85 (0.85)
4e35	ANSRWPTSIL	10	1-10	1.59 (1.59)	1.27 (1.27)
4esg	EPPLNPHGSARAEVHL	16	1-16	4 (4)	3.33 (3.33)
4ezn	SYLPRP	6	1-6	0.66 (0.66)	0.51 (0.51)
4ezo	PPYLPRPR	8	1-8	0.85 (0.85)	0.43 (0.43)
4f14	PPPTLPKPKLP	11	1-11	2.17 (2.17)	1.44 (1.44)
4faj	LVTLVFV	7	1-7	2.48 (2.48)	0.83 (0.83)
4g4l	GNADEYKEEDQERRKRKKRSG	21	1-21	4.11 (4.11)	3.73 (3.73)
4g8i	KRWIIMGLNK	10	1-10	1.72 (1.72)	0.97 (0.97)
4hrg	MKIPKFTF	8	1-8	1.25 (1.25)	0.83 (0.83)
4hsu	DPHFHHFLLSQT	12	1-12	3.8 (3.8)	3.2 (3.2)
4i4w	ILAKFLHRL	9	1-9	3.1 (3.1)	1.67 (1.67)
4j77	KEKSD	5	1-5	2.04 (2.04)	1.26 (1.26)
4j78	KTKLL	5	1-5	2.4 (2.4)	1.23 (1.23)
4j82	KSHQE	5	1-5	2.39 (2.39)	1.13 (1.13)
4j86	TFKKTN	6	1-6	2.86 (2.86)	2.15 (2.15)
4j8s	RRLPIFNRISVS	12	1-12	2.73 (2.73)	2.63 (2.63)
4k38	TSPMCAPARSM	11	1-11	3.89 (3.89)	2.84 (2.84)
4odm	MKPFIF	6	1-6	2.47 (2.47)	1.41 (1.41)
4odm	NPKMKPFIFGA	11	1-11	2.47 (2.52)	1.99 (1.99)
4rtw	APPLPPRNRPRL	12	1-12	1.65 (1.65)	1.04 (1.04)
4tut	GGYMLG	6	1-6	1.01 (1.01)	0.61 (0.61)
4yxb	VFQQLG	6	1-6	1.51 (1.51)	1.14 (1.14)
5mxt	WYHRLSHIHSRLQD	14	2-14	0.76 (0.65)	0.46 (0.34)
5v0y	GFCWYVCVYRNGVRVCYRRCN	21	1-21	1.26 (1.26)	1.1 (1.1)
5wlx	GDCHKFLGWCRGEKDPCCEHLTCHVKHGWCVWDGTI	36	1-34	2.37 (2.31)	2.2 (2.09)
5wrx	VARGWGRKCPLFG	13	1-13	3.43 (3.43)	3.02 (3.02)
5x1g	KRDKIKEEEQKKKEWINQERQKTLQRLRSFK	31	1-31	5.51 (5.51)	4.47 (4.47)
5xa6	GFGCPFNQGKCHRHCRSIRRRGGYCDGFLKQRCVCYRK	38	2-37	3.07 (2.39)	2.8 (2.32)
5zgd	GFGGNDNFG	9	1-9	2.78 (2.78)	2.33 (2.33)
6a5j	IKKILSKIKKLLK	13	1-13	2.68 (2.68)	2.02 (2.02)
6atn	GSVFINVKCRGSPECLPKCKEAIGKSAGKCMNGKCKCYP	39	1-39	1.39 (1.39)	1.28 (1.28)
6awm	GLLGITD	7	1-7	3.77 (3.77)	2.2 (2.2)
6bb6	GMACQFWSCNSSCISRGYRQGKCWGIQYKYCQCY	34	5-23,30-34	1.94 (1.42)	1.94 (1.31)
6cli	GSNQNNF	7	1-7	1.31 (1.31)	1.24 (1.24)
6dri	EHCADEKNFDCRRSLRNGDCDNDDKLLEMGYYCPVTCGFC	40	3-40	3.14 (2.91)	2.81 (2.51)
6fgm	ACFLTRLGTYVC	12	1-12	2.17 (2.17)	1.69 (1.69)
6g4i	FLPILASLAAKFGPKLFCLVTKKC	24	2-24	5.11 (5.1)	3.65 (3.66)
6g9g	TASNSS	6	1-6	1.48 (1.48)	1.11 (1.11)
6gs9	GLFDIVKKVVGAFGSL	16	2-11	0.91 (0.38)	0.8 (0.26)
6gwx	PPKKPKKPGDNATPEKLAAYEKELAAYEKELAAY	34	1-9,11-34	1.9 (1.88)	1.75 (1.74)
6jic	QDDPTCGKPCNTMDDCSNGWFCQACWNSRKTCGPFV	36	1-35	2.68 (2.66)	2.55 (2.47)
6mbm	KWAVRIIRKFIKGFIS	16	2-15	1.15 (0.9)	0.9 (0.75)
6mi9	PMARNKILGKILRKIAAFK	19	2-19	0.71 (0.58)	0.65 (0.52)
6mzt	GDIKCSGTRQCWGPCKKQTTCTNSKCMNGKCKCYGCV	37	1-37	1.77 (1.77)	1.27 (1.27)
6nug	CDMEVSCPDGYTCCRLQSGAWGCC	24	5-16,18-20,23	1.66 (1.49)	1.52 (1.3)
6o8j	AGKETIRQYLKNEIKKKGRKAVIAW	25	1-25	4.14 (4.14)	3.52 (3.52)

6oqp	SKWICANRSVCPI	13	1-13	2.17 (2.17)	1.8 (1.8)
6pin	KWCFRVCYRGICYRRCRG	18	1-18	1.4 (1.4)	1.19 (1.19)
6q8l	RPLDTVQRPKGY	12	1-12	4.23 (4.23)	2.88 (2.88)
6r9z	EVNPPVP	7	1-7	1.68 (1.68)	0.96 (0.96)
6rqs	RRWRRWWRRWWRRWRR	16	1-16	1.11 (1.11)	0.99 (0.99)
6rrl	FRIMRILRVLKL	12	1-11	1.18 (1.04)	0.97 (0.93)
6s6m	FKRIVQRIKDFLR	13	1-13	0.68 (0.68)	0.5 (0.5)
6saa	RCLHAGAACSGPIQKIPCCGTCSRRKCT	28	1-28	2.08 (2.08)	1.94 (1.94)
6twg	FLPKILRKIVRAL	13	1-13	1.77 (1.77)	0.94 (0.94)
6u7u	GRATKSIPPRAFPD	14	1-14	2.02 (2.02)	1.23 (1.23)
6uow	AVAAGA	6	1-6	1.59 (1.59)	1.21 (1.21)

Table 2: Performance comparison of APPTEST, PEP-FOLD 3.5 and PEPstrMOD B-RMSD values on peptides with 9-25 amino acids. Numbers in brackets are B-RMSD values for the peptides' rigid cores only. On average, APPTEST produces more native structures than both PEP-FOLD and PEPstrMOD.

PDB	L	RC	APPTEST PRIME	PEP-FOLD PRIME	PEPstrMOD ONLY	APPTEST BEST	PEP-FOLD BEST
1a13	14	2-14	2.26 (2.05)	2.05 (1.85)	3.01 (2.98)	2.02 (1.89)	1.82 (1.73)
1b03A	18	-	5.05	2.69	4.26	2.85	2.33
1du1	20	-	4.94	5.12	5.63	4.16	4.88
1e0q	17	-	3.77	2.31	4.54	2.79	0.98
1egs	9	-	6.52	2.04	2.08	3.65	1.05
1gjf	14	4-14	1.86 (0.78)	2.38 (0.73)	6.53 (5.28)	0.96 (0.55)	1.39 (0.66)
1in3	12	-	1.24	1.67	2.50	1.20	1.55
1l2y	20	-	1.59	2.90	6.24	1.26 (1.26)	1.18
1l3q	12	-	5.98	3.92	3.14	3.80 (3.80)	2.57
1lcx	13	3-12	2.25 (1.20)	2.51 (1.46)	4.30 (3.15)	1.80 (1.12)	2.42 (1.38)
1niz	14	-	2.32	1.68	5.55	1.74	1.22
1nkf	16	-	5.41	4.71	4.26	4.13	1.56
1pef	18	-	0.60	0.90	0.99	0.60	0.69
1rpv	17	4-16	0.86 (0.35)	0.74 (0.54)	5.57 (5.46)	0.63 (0.28)	0.73 (0.38)
2bta	15	4-9	4.53 (2.61)	4.50 (2.60)	4.17 (3.26)	3.08 (1.70)	4.36 (2.51)
1c98	10	-	2.84	5.39	4.63	2.30	2.60
1d6x	13	1-12	2.53 (2.50)	2.94 (2.94)	4.59 (4.51)	2.34 (2.16)	2.29 (2.20)
1d7n	14	2-13	1.08 (0.69)	1.04 (0.64)	2.88 (2.23)	0.89 (0.61)	0.97 (0.62)
1d9j	20	10-20	2.06 (0.89)	4.38 (1.18)	6.12 (4.03)	1.50 (0.61)	2.58 (0.98)
1d9l	17	4-15	1.47 (0.47)	1.71 (0.67)	5.83 (4.93)	1.25 (0.39)	1.67 (0.62)
1d9m	18	10-18	1.95 (1.31)	2.62 (1.16)	4.38 (3.46)	1.66 (0.58)	2.27 (0.97)
1d9o	20	11-20	3.17 (0.44)	3.10 (0.51)	5.23 (3.17)	2.47 (0.34)	2.78 (0.43)
1d9p	20	10-20	1.88 (0.54)	2.18 (0.63)	3.52 (3.33)	1.62 (0.46)	1.92 (0.57)
1dn3	15	-	1.10	1.14	7.20	1.02 (	0.91
1g89	13	1-12	2.55 (2.34)	4.51 (4.36)	3.15 (3.13)	1.82 (1.75)	3.28 (3.15)
1hu5	18	2-18	1.96 (1.79)	1.60 (1.54)	2.49 (2.45)	1.61 (1.48)	1.39 (1.33)
1hu6	18	2-18	3.26 (3.25)	3.43 (3.43)	4.26 (4.09)	2.51 (2.50)	2.71 (2.43)
1hu7	18	2-18	2.05 (1.94)	1.79 (1.63)	3.33 (3.31)	1.77 (1.69)	1.79 (1.58)
1id6	15	1-14	5.54 (5.59)	5.50 (5.55)	7.42 (7.4)	4.96 (5.02)	4.50 (4.48)
1jav	19	-	1.45	6.13	7.44	1.43	3.28
1kzv	18	2-18	1.20 (1.02)	1.78 (1.65)	4.73 (4.34)	0.79 (0.72)	1.57 (1.44)
1m02	12	2-11	4.23 (3.13)	4.30 (3.33)	5.89 (4.30)	2.39 (1.86)	3.01 (2.75)
1myu	12	-	1.73	3.00	4.38	1.22	0.96
1odp	20	-	1.83	1.89	4.75	1.34	1.75
1p0j	19	2-19	1.58 (1.30)	1.72 (1.57)	4.04 (3.92)	1.27 (1.11)	1.57 (1.39)
1p0l	19	2-19	1.66 (1.28)	1.88 (1.59)	5.12 (5.22)	1.27 (1.15)	1.69 (1.49)
1p0o	19	2-19	1.54 (1.34)	1.98 (1.87)	4.77 (4.61)	1.29 (1.06)	1.65 (1.52)
1p5k	19	2-18	1.29 (1.03)	1.77 (1.54)	4.74 (4.16)	1.21 (0.72)	1.62 (1.35)
1qcm	11	2-11	2.95 (2.99)	2.49 (1.90)	3.92 (3.71)	2.72 (2.69)	1.10 (1.02)
1qfa	13	-	1.01	0.72	5.34	0.90	0.66
1sol	20	-	4.14	3.27	6.38	2.56	2.59
2bp4	16	1-15	2.17 (1.64)	5.45 (5.21)	6.31 (6.15)	1.64 (1.60)	4.12 (4.06)
MEAN			2.60 (2.24)	2.81 (2.38)	4.66 (4.33)	1.96 (1.69)	2.05 (1.71)

PDB: Protein Data Bank identifier. L: peptide length. RC: rigid core. B-RMSD: Backbone root mean square deviation. Results are reported for the best model and the first model, for both the full structure and the rigid core. APPTEST first model is that with the lowest XPLOR-NIH energy. PEP-FOLD first model is the one with the lowest sOPEP energy value. PEPstrMOD only returns a single structure. Numbers within brackets are rigid core B-RMSDs, where applicable.

Table 3: Performance comparison of APPTEST, PEP-FOLD 3.5 and PEPstrMOD B-RMSD values on peptides with 26-40 amino acids. Numbers in brackets are B-RMSD values for the peptides' rigid cores only. On average, APPTEST-predicted structures are more native than those predicted by PEP-FOLD, especially in the case of the lowest-energy prime structures.

			APPTEST	PEP-FOLD	APPTEST	PEP-FOLD
PDB	L	RC	PRIME		BEST	
1by0	27	1-23	1.68 (0.98)	1.65 (1.38)	1.42 (0.96)	1.37 (1.22)
1yyb	26	1-20	2.65 (1.57)	3.26 (1.29)	1.89 (1.03)	1.85 (1.00)
2kbl	27	6-27	2.35 (2.43)	7.50 (5.79)	1.92 (1.87)	3.35 (2.14)
2k76	30	4-29	9.91 (6.75)	4.40 (3.77)	5.95 (4.46)	1.29 (1.03)
2gdl	31	8,10-11,14-15,21-29	6.32 (4.90)	7.36 (4.69)	4.94 (4.07)	5.01 (4.14)
2l0g	32	5-32	3.73 (1.82)	3.57 (1.48)	2.27 (1.36)	1.70 (1.04)
2bn6	33	4-29	2.17 (1.01)	12.44 (9.69)	1.62 (0.95)	8.32 (6.42)
2ovc	30	-	2.89	10.39	2.40	7.16
1bwx	39	15-28	7.06 (0.90)	4.31 (1.45)	5.24 (0.74)	4.06 (1.42)
2kya	34	11-30	3.37 (1.25)	8.52 (6.61)	3.09 (0.97)	6.74 (4.35)
1wy3	35	1-26	1.39 (1.44)	4.26 (3.58)	1.35 (1.32)	3.18 (2.60)
1wr3	36	5-15,17-34	1.47 (1.26)	5.01 (3.39)	1.47 (1.26)	2.61 (1.80)
1wr4	36	5-34	7.36 (6.67)	5.36 (3.67)	2.22 (1.64)	2.08 (1.67)
2ki0	36	5-11,13-36	11.92 (10.82)	2.51 (1.89)	8.38 (8.48)	2.18 (1.80)
1e0m	37	3-35	2.75 (2.32)	4.16 (4.20)	2.07 (1.79)	2.51 (2.33)
1e0n	27	1-25	6.85 (6.42)	6.79 (6.29)	3.73 (3.75)	5.10 (5.19)
1yiu	37	5-36	2.11 (1.86)	5.67 (4.91)	2.00 (1.61)	2.90 (2.40)
1bhi	38	7-11,14-33	4.02 (1.74)	7.26 (4.78)	3.19 (1.40)	4.44 (2.83)
1i6c	39	2-11,16-33	4.52 (2.03)	6.98 (4.32)	3.68 (1.92)	3.57 (1.75)
1jrj	39	11-38	2.22 (1.31)	3.72 (2.71)	2.06 (1.23)	3.06 (2.57)
2ysc	39	8-39	5.03 (4.81)	5.96 (4.98)	4.07 (3.89)	3.76 (2.57)
1e0l	37	6-37	2.87 (1.83)	5.13 (3.78)	2.71 (1.83)	3.61 (2.41)
2ysf	40	7-39	4.67 (3.59)	6.98 (5.08)	3.86 (3.56)	3.77 (2.29)
2ysg	40	7-38	8.99 (8.32)	6.32 (4.68)	3.89 (3.15)	3.42 (2.24)
2ysh	40	7-37	4.09 (2.74)	6.18 (3.74)	3.44 (1.99)	3.51 (2.23)
2ysi	40	9-40	4.42 (3.52)	8.64 (7.92)	4.19 (3.35)	3.77 (2.86)
1ywj	28	-	2.03	3.81	1.92	2.09
1ymz	37	3-13,5-32	2.37 (1.48)	5.07 (3.50)	2.10 (1.30)	2.57 (1.68)
3e21	40	-	9.14	2.98	6.95	2.98
1use	40	-	3.05	2.15	2.10	1.16
MEAN			4.45 (3.36)	5.61 (4.30)	3.20 (2.44)	3.43 (2.58)

PDB: Protein Data Bank identifier. L: peptide length. RC: rigid core. B-RMSD: Backbone root mean square deviation. Results are reported for the best model and the first model, for both the full structure and the rigid core. APPTEST first model is that with the lowest XPLOR-NIH energy. PEP-FOLD first model is the one with the lowest sOPEP energy value. Numbers within brackets are rigid core B-RMSDs, where applicable.

Table 4: Performance comparison of APPTEST, PEP-FOLD 2.0, Peplook and PEPstr-MOD B-RMSD values on cyclic peptides. Numbers in brackets are B-RMSD values for the peptides' rigid cores only. APPTEST produces significantly more native-like structures than PEP-FOLD, Peplook and PEPstrMOD, with APPTEST's mean lowest energy structure B-RMSD being just 64% that of the next best, PEP-FOLD.

PDB	L	RC	APPTEST	PEP-FOLD PRIME	PEPLOOK	PEPstrMOD ONLY	APPTEST	PEP-FOLD BEST	PEPLOOK
1n0c	10	-	0.74	0.68	3.2	2.95	0.59	0.64	3.1
1n0a	11	-	2.38	0.57	2.8	3.57	0.9	0.55	2.8
1etl	12	-	2.05	3.28	2	2.89	1.33	1.67	2
1im1	12	-	1.46	2.12		3.12	0.98	1.35	
1gnb	13	2-7	2.44 (1.91)	3.72 (2.75)	2.4	4.07 (2.10)	1.97 (1.32)	2.78 (1.49)	2.4
1hje	13	-	1.43	4.24	3.7	3.35	1.41	2.32	3.7
1im7	13	-	3.15	4.34		4.30	2.08	2.63	
1xgb	13	2-13	2.70 (2.67)	3.02 (3.02)	(2.4)	3.41 (3.37)	1.75 (1.68)	2.56 (2.56)	(2.4)
2i28	13	-	2.14	1.51	2.1	3.57	1.65	1.51	2.1
1b45	14	2-9,13-14	1.75 (1.21)	3.85 (3.47)	2.2	4.96 (4.16)	1.36 (1.13)	1.35 (1.14)	2.2
1jbl	14	-	1.46	2.09	3.2	3.37	1.19	2.00	3.2
1r8t	15	1-14	1.89 (1.79)	3.55 (3.59)	2.7	2.40 (2.27)	1.68 (1.56)	2.28 (2.33)	2.7
1kwd	16	2-12,14-15	2.56 (2.30)	2.54 (2.03)	3.0	3.27 (2.72)	1.93 (1.57)	1.53 (1.24)	3 ()
1mii	16	-	1.17	4.25	3.2	3.53	1.1	1.21	3.2
2efz	16	-	5.03	3.07	3.4	5.30	1.75	2.06	3.4
1nim	17	5,7-8,10-14	3.14 (2.48)	3.87 (2.8)	3.7	4.82 (3.68)	2.42 (1.54)	3.04 (2.1)	2.9
1ien	19	2-19	2.35 (1.87)	4.99 (4.71)	3.3	5.81 (5.64)	1.56 (1.45)	2.30 (2.04)	3.3
1x7k	19	8-9,14,16-18	3.63 (1.97)	4.95 (3.31)	3.6	4.93 (3.66)	2.17 (1.52)	4.37 (2.49)	3.6
1kcn	21	3-9,14-21	2.72 (2.46)	6.39 (6.64)	4.7	4.79 (4.63)	2.41 (2.08)	2.69 (2.44)	4.7
1rpc	21	1-11,13-19	1.54 (1.53)	5.92 (5.86)	6.5	5.85 (5.94)	1.54 (1.53)	4.60 (4.23)	6.5
1ter	21	2-21	4.33 (3.77)	5.32 (5.36)	3.7	4.57 (4.51)	4.33 (3.77)	2.89 (2.55)	3.7
1v6r	21	-	4.99	5.69	5.8	6.98	3.87	3.56	4.7
1hp9	22	-	1.91	2.97	5.4	5.86	1.9	2.14	4.3
2ajw	22	1-2,5-13,17	1.79 (0.64)	3.01 (1.67)	(2.4)	4.54 (3.49)	1.55 (0.60)	1.75 (0.72)	(2.4)
1oig	24	2-11,14-23	2.98 (2.49)	6.11 (5.04)	6.4	7.02 (6.72)	2.80 (2.32)	4.38 (4.00)	6.4
1orx	24	-	1.19	4.1	6.8	7.39	1.13	3.16	6.8
1sp7	24	-	3.20 (2.96)	6.87	4.6	6.81	2.61 (2.41)	2.56	4.6
2oq9	24	8-22	7.89 (4.09)	7.82 (3.56)	(3.8)	6.01 (4.01)	5.04 (2.76)	6.16 (3.45)	(3.8)
1wqc	26	-	3.08 (1.21)	2.12	4.8		2.25 (1.21)	1.43	4.7
1v5a	28	1-9,12,14,15,17-28	1.81 (1.78)	6.25 (6.37)	5.4		1.74 (1.70)	3.45 (3.58)	5.1
1wm8	28	-	2.3	6.67	5.7		2.21	4.43	5.7
2it7	28	-	1.67	4.02	6.7		1.46	3.13	5.5
2nx7	28	2-28	5.94 (6.04)	6.59 (6.63)	7.4		3.52 (3.53)	5.12 (4.92)	6.2
1mmc	30	3-29	2.36 (2.28)	5.49 (5.28)	(5.0)		1.71 (1.64)	4.41 (3.96)	(4.4)
MEAN (10-25)			2.64 (2.26)	3.96 (3.56)	3.84*	4.62 (4.29)	1.96 (1.68)	2.50 (2.14)	3.71*
MEAN (ALL)			2.68 (2.09)	4.18 (3.81)	4.23*		1.99 (1.55)	2.71 (2.37)	4.02*

PDB: Protein Data Bank identifier. L: peptide length. RC: rigid core. B-RMSD: Backbone root mean square deviation. Results are reported for the best model and the first model, for both the full structure and the rigid core. APPTEST first model is that with the lowest XPLOR-NIH energy. PEP-FOLD first model is the one with the lowest sOPEP energy value. PEPstrMOD only returns a single structure. Numbers within brackets are rigid core B-RMSDs, where applicable. Peplook results are taken from Beaufays et al. (2012).
